# Vocational rehabilitative decisions after symptoms and findings consistent with hand-arm vibration syndrome in the Swedish surveillance system – a mixed-method design

**DOI:** 10.1186/s12995-024-00432-7

**Published:** 2024-08-12

**Authors:** Frida Thorsén, Catarina Nordander, Carl Antonson

**Affiliations:** 1 Center for Primary Health Care Research, Department of Clinical Sciences Malmö, Lund University, Malmö, Sweden; 2 Division of Occupational and Environmental Medicine, Department of Laboratory Medicine, Lund University, Scheelevägen 8, 223 63 Lund, Sweden

**Keywords:** Hand-arm vibration syndrome, Surveillance system, Rehabilitation, Underestimation, Neurologic examination, Qualitative research

## Abstract

**Background:**

EU workers exposed to hand-arm vibration should be offered health surveillance to detect early symptoms, and findings, of Hand-Arm Vibration Syndrome (HAVS). To execute the mandatory vocational rehabilitation, the employer needs to be aware of injuries found in the medical check-up. We aimed to analyse: 1) How physicians graded the neurosensory component of HAVS on the Stockholm Workshop Scale (SWS), compared to semi-objective findings. 2) What vocational rehabilitative decisions (VRD) were taken by physicians after examinations. 3) Whether the VRDs differed in relation to the SWS-grading.

**Methods:**

Data came from 660 medical records - all examinations performed during twelve consecutive months in one large Swedish occupational healthcare company. 572 individuals had data on the SWS from the physician. For the qualitative analysis, we used the inductive-iterative immersion-crystallization method.

**Results:**

60% of the examined workers had symptoms and 32% had semi-objective findings consistent with HAVS. The physicians’ SWS gradings were underestimated in 59% of the cases with semi-objective findings. The VRDs were classified, relative to communication with the employer, as: “**Adequate**” (57%), when no injury was present, communication had already taken place, was planned, or was no longer needed in the absence of further exposure, “**Semi-adequate”** (18%), if no plan for communication was yet established or only communicated through a document with a shorter time until next check-up, and “**Inadequate**”(25%), when patients refused (20%), or physicians failed to communicate with the employer, despite findings (80%). Underestimated SWS-gradings of HAVS were significantly associated with more “Inadequate” VRDs in the group with semi-objective findings.

**Conclusions:**

Occupational physicians underestimate the number of individuals with SWS 2–3 compared with semi-objective findings and regularly fail to communicate to the employer despite findings of HAVS. The underestimation of SWS-grading, followed by inadequate VRDs, excludes many workers from the employer’s mandatory protective measures which may lead to aggravation of an untreatable injury in the affected individual and development of HAVS in their similarly exposed colleagues.

## Background

For over 100 years, exposure to hand-arm vibration has been known to cause injuries in hands and arms [1]. Today, this is referred to as Hand-Arm Vibration Syndrome (HAVS), a disorder of injury to nerves and vessels that worsens with increased exposure time and amplitude of vibrations [2]. Prevention is mandated as HAVS in the more advanced stages is considered a chronic condition, only reversible to a small extent in the case of vessel injuries and virtually irreversible in the case of nerve injuries [3–6].

EU workers exposed to hand-arm vibration should be offered health surveillance to detect early symptoms, and findings, of plausible HAVS [7]. In Sweden, this is declared in the provision AFS 2019:3 [8], which states that companies with employees exposed to hand-arm vibrations have to provide Medical Check-ups in Work-life (MCV) for vibrations. The employer is also responsible for vocational rehabilitation, regulated by the Work Environment Act (Ch. 29, § 2), whereas the health care system is responsible for medical rehabilitation. Most large companies in Sweden use an Occupational Healthcare Service (OHS) provider to perform MCVs and ensure the medical quality of vocational rehabilitation.

The ways that physicians manage results from the MCV can be divided into different Vocational Rehabilitative Decisions (VRDs). If symptoms, or findings, of HAVS are detected at an MCV, it is desirable that the employer is informed about the result. If not, the appropriate mandatory vocational rehabilitation cannot take place. Thus, if an employer is unable to do this because of insufficient communication after the MCV, the VRD must be considered inadequate. Due to laws on professional secrecy, the physician is only allowed to present the results from the MCV if the employee gives their permission. Therefore, some VRDs may be inadequate due to the employee’s decision, and others due to that of the physician. OHSs often recommend the results from the MCV be communicated through a rehabilitation meeting with the employer, employee, and occupational physician present (also often including a safety representative as well as a human-resources representative), which makes for a good common ground of understanding in vocational rehabilitation. In this meeting, the occupational physician informs about the employee’s medical status and rehabilitative options, divided into medical rehabilitation and work-life rehabilitation. Initially, vocational rehabilitation focuses on ways to adapt the work situation to the needs of the injured worker. If no adaptations can be made, the employer is responsible for examining the possibilities of redeployment within the company before any termination of employment can take place. Since no medical treatment for HAVS is known [3–6], vocational rehabilitation is normally the only remaining way for a physician to help the patient.

Both the long-term prognosis and the recommendations for vocational rehabilitation are based on the grade of injury according to the Stockholm Workshop Scale (SWS) that has been used since the 1980s [4, 9–12]. The SWS is a semi-objective scale as it depends on the examined individuals reporting their symptoms, and response to neurological stimuli. Modified and alternative scales have been proposed, but so far SWS is considered the gold standard [6, 10, 13]. Symptoms and findings for injuries of both vascular (finger blanching) [14] and sensorineural [15] character can be graded. The sensorineural scale has four stages (SWS SN 0–3) based on symptoms of numbness with or without tingling (SN1), sensory deficiency (SN2), and impaired fine motor skills (SN3). The progression of HAVS is assumed to follow these stages, although individuals can, for example, develop findings of sensorineural deficits without reporting present or previous symptoms [13]. Importantly, the SWS-scale relies on the correct interpretations of the reported clinical findings by the physician. There are Swedish guidelines that well describe the SWS-grading that are recommended by the branch organization as well as many OHSs [16, 17]. An error in the SWS-grading could be caused by an incorrect interpretation of the examination (e.g. the inability to perceive a Temproll as warm that should be graded SWS SN 2 is incorrectly graded as SWS SN 1). To our knowledge, no inter-rater variability assessment has been published on the SWS, nor any evaluation of the quality of the assessments in clinical material.

Remarkably, a Swedish report [18] revealed that the SWS grading presented in medical records was not necessarily the same as the grade that the actual symptoms and findings from the medical record would objectively translate to on the SWS sensorineural [15]. It was reported that clinical findings of obvious sensory deficiency sometimes were considered “normal findings”, despite the recommendations in the Swedish guidelines [16, 17]. Despite the patients having sensory perception deficiency, neurological injuries with semi-objective findings were often graded with an SWS below two [18], the cut-off below which no clinical findings are present [15]. At an SWS grading of two or above, a total abstinence of exposure is recommended and vocational rehabilitation should be initiated [11, 19].

There is still a great lack of knowledge concerning how occupational physicians manage findings at the MCV and whether individuals with incipient HAVS get their crucial vocational rehabilitation so that exposure can be reduced.

Our aims were to:

1. Examine how occupational physicians graded the neurosensory component of HAVS by the Stockholm Workshop Scale (SWS) and evaluate the accurateness of these SWS gradings compared with semi-objective findings.
2. Analyse which vocational rehabilitative decisions (VRD) occupational physicians took at their MCV.
3. Examine whether there was a difference between the VRDs in terms of how much the SWS grading differed when graded by the occupational physicians as compared to the semi-objective grading.

## Methods

Data were collected from the computerised medical records program CGM J4 (Compugroup Medical Sweden AB, Sweden), where the medical records of all MCVs from all clinics in Sweden at the OHS Feelgood were stored. We collected data from all MCVs performed between 6th March 2019 and 6th March 2020. Feelgood is one of the three largest OHSs in Sweden and has as customers several of the largest construction companies in Sweden alongside many companies in the manufacturing industry, both with heavily hand-arm vibration exposed workers. The clinics are situated all over Sweden.

We found 660 unique individuals with an MCV registered in their medical records. 619 (94%) were male and 41 (6%) were female (legal sex), the median age was 48 (IQR 21, range 19–67). This population was used for the qualitative analysis of VRDs.

To evaluate the accuracy of the SWS grading, only individuals with a SWS grading recorded by the physician were included. This left 572 of the original population: 534 (93%) men and 38 (7%) women. The median age did not change.

To evaluate grading of the neurosensory component of HAVS we extracted data from the medical records where SWS sensorineural (SN) [15] was graded by the physician, i.e. the original SWS-grading (SWS-Orig).

Further, we graded the stage of HAVS using semi-objective findings from the standardised four tests that are recommended for SWS grading in the national guidelines [16] when the results from these were recorded in medical records. The grading is shown in Table 1 and the interpretation of the findings could be considered gold standard [16]. If one of the four examined fingers (dig. II and V bilaterally) had at least one finding this gave the semi-objective SWS SN (SWS-SO). If there was no data on semi-objective findings, the original SWS-grading (SWS-Orig) was used for SWS-SO. If symptoms of HAVS, but no findings of sensorineural deficit, were noted in the medical record, it was graded SWS-SO 1. If diminished manipulative dexterity was noted, it was graded SWS-SO 3. When a justification for the grading was presented in the medical record, e.g. the sensorineural deficit was due to an injury from a cut, SWS-Orig was also used for SWS-SO.

**Table 1:** Cut-off values and gradings for Stockholm Workshop Scale SN for semi-objective testing (SWS-SO)

Test method	Result	Result interpretation^#^	SWS-SO
Semmes Weinstein	2.81 (Green)	Normal	0
Monofilament	3.61 (Blue)	Quasi normal	0
	4.31 (Purple)	Diminished light touch	2
	≥ 4.56 (Red)	Diminished protective sensation	2
Static two-point discrimination	> 5 mm	Diminished tactile spatial acuity	3
Tuning fork (128 Hz)	Reported diminished	Diminished vibration sensation	2
Rolltemp (25 °C and 40 °C)	Reported diminished	Diminished thermal sensation	2

^**#**^Interpretation threshold values from the Swedish national guidelines [16] considered gold standard

For the qualitative analysis, we used the inductive-iterative immersion-crystallization method as the data sources were already condensed from the longer interview, the anamnesis of medical appointment, in the form of a medical record, to which all the authors are very familiar [20, 21]. All medical records were read by the last author. After a first read-through, ten VRDs were defined together with the first author. The last author then read through all the records again and a VRD was defined for every individual. The unclear cases were discussed with the other authors.

To increase the trustworthiness and facilitate new clues and insights into the entirety of the research objective, we presented the data from the qualitative method in quantitative tables for the mixed method analysis [22, 23].

For workers with a SWS-SO grading of one or more, we defined a rehabilitative decision with respect to communication on a three-tier scale: “Inadequate” if no communication took place, “Semi-adequate” if the communication did not include diagnosis, and “Adequate” if the communication was not needed or took place. “Inadequate” was further subdivided into whether the patient or the physician took the decision. For workers with SWS SO 0 all communication was considered adequate since no vocational rehabilitation, and thus no communication, was needed, see below.

### Statistics

We used STATA MP 17.0 (StataCorp, Texas, USA) for the statistical calculations.

Agreement between the SWS-Orig and the SWS-SO grading was estimated by Cohen’s kappa statistic. A misclassification of the grading was considered worse if a worker was graded as SWS-Orig 0 or 1 but was semi-objectively reclassified as SWS-SO 2 or 3. A linearly weighted kappa was used to adjust for the severity of misclassification.

The number of false negatives was calculated, i.e. the number of individuals with an SWS-Orig 0–1 that changed to a SWS-SO 2–3. The proportion of false negatives was estimated, together with a 95% exact binomial confidence interval.

For the patients that were graded SWS-SO 2–3, the Chi-square test was used to examine the association between the SWS SN and the occupational physicians grading the rehabilitative action categorised regarding communication to the employer into adequate, semi-adequate and inadequate.

## Results

### Agreement between the scales

In 572 individuals we compared gradings for Sensorineural SWS-SO with those for SWS-Orig (Table 2). According to the semi-objective testing, 40% showed no findings (SWS-SO SN 0), 28% had symptoms only (SWS SN 1), and 32% had semi-objective findings of possible sensorineural injury (SWS SN 2 or 3). The number of individuals with SWS-SO 2–3 almost doubled compared with the SWS-Orig. The increase was largest (sixfold) in the most severe stratum (SWS SN 3).

**Table 2:** Stockholm Workshop Scale (SWS) sensorineural disturbance grading by the occupational physicians (SWS-Orig) and semi-objectively (SWS-SO)

Outcome	SWS-Orig	SWS-SO
SWS Sensorineural 0, n (%)	275 (48)	226 (40)
SWS Sensorineural 1, n (%)	215 (38)	162 (28)
SWS Sensorineural 2, n (%)	72 (13)	122 (21)
SWS Sensorineural 3, n (%)	10 (2)	62 (11)
Total	572 (100)	572 (100)

One hundred and eight individuals with SWS-Orig 0–1 (without clinical findings) had SWS-SO 2–3 (19%; 95% CI 16–22%, Table 3). The agreement between the SWS-Orig and SWS-SO as estimated by Cohen’s kappa was 0.57. Weighted kappa, which takes into account the severity of misclassification, was 0.50.

**Table 3:** Stockholm Workshop Scale (SWS) sensorineural disturbance grading by the occupational physicians (SWS-Orig) and semi-objectively (SWS-SO)

	SWS-SO 0–1 *n* (%)	SWS-SO 2–3 *n* (%)
SWS-Orig 0–1	382 (67)	108 (19)
SWS-Orig 2–3	6 (1)	76 (13)

### Vocational rehabilitative decisions

In 659 of the 660 individuals the VRD could be deduced from the medical record. Then, alongside the four default RAs in the medical records (*No rehabilitation meeting needed*, *Rehabilitation meeting accepted*, *Rehabilitation Meeting declined*, and *No data*), we found seven new differing categories of VRDs. Ordered from most adequate to most inadequate with respect to communication with the employer, the new categories were:

1. “*Employee no longer exposed*”, where individuals were no longer exposed to hand-arm vibrations due to retirement or a new job description without exposure.
2. “*Alternative follow-up*”, where there had been communication with the employer but without the patient, implying a lower grade of transparency in the process.
3. “*Already known HAVS*”, where the individuals already had a HAVS-diagnosis known by the employer but were still sent to MCV.
4. “*Medical investigation commenced*”, where a medical investigation had commenced, primarily through a referral to a public health service usually a clinic specialized in occupational and environmental medicine, orthopedics or hand surgery, primary health care or imaging diagnostics. Importantly, no contact with the employer had yet been noted in the clinical record.
5. “*Shorter period until next MCV*”, where the formalized document given to all employees to be transferred to the employer stipulated less than the normal three years until the next MCV. This is an indication to the employer that there is something deemed to increase the risk for HAVS, but where no other information than the shorter interval is transferred to the employer.
6. “*Individual recommendations only*”, where the physician gave individualised recommendations on how to reduce exposure to vibrations but no data on communication with the employer was noted.
7. “*No action despite findings*”, where the physician recorded sensorineural findings or finger-blanching in accordance with HAVS but no intervention, nor rehabilitation, was initiated.

The VRDs were hierarchically ordered by number so a combination of an *Alternative follow-up* (VRD no 2) and a document with a *Shorter period until next MCV* (VRD no 5) was classified as an *Alternative follow-up.*

The VRDs were classified, with respect to communication, as: “**Adequate**”(57% of the total population), when no injury was present (*No rehab needed*), communication had already taken place (*Already known HAVS* or *Alternative follow-up*), was planned (*Rehabilitation accepted)*, or was no longer needed in absence of further exposure (*Employee no longer exposed*), “**Semi-adequate”** (18%), if no plan for communicating was yet established (*Medical investigation commenced*) or only communicated through a document with shorter time until next check-up (*Shorter period until next MCV*), and “**Inadequate**”(25%), when patients refused (*Rehabilitation declined*), or physicians failed to communicate, despite findings *(No action despite findings or Individual recommendations only*). Table 4 Gives an overview of the different VRDs in relation to adequacy and the decision’s agent.

**Table 4:** Overview of the vocational rehabilitative decisions (VRDs) in relation to adequacy and the decision’s agent

Adequacy of VRD with respect to communication with employer:		VRD taken by:		
		Physician	Employee	Physician and Employee
***Adequate*** Not needed, accepted, planned, or already communicated		*No rehab needed* *Employee no longer exposed* *Alternative follow-up*	N/A	*Already known HAVS* *Rehabilitation accepted*
***Semi-adequate*** Not yet communicated or communicated without diagnosis		*Shorter period until next MCV*	N/A	*Medical investigation commenced*
***Inadequate*** Not communicated at all		*Individual recommendations only* *No action despite findings*	*Rehabilitation declined*	N/A

### Vocational rehabilitative decisions in relation to SWS-grading

In the group with SWS-SO 1–3, i.e. the 323 individuals with a need of VR, 25% had an Adequate VRD, 31% a Semi-adequate VRD, and 45% an Inadequate VRD, whereof 36% were not communicated due to the physician (Table 5).

**Table 5:** Vocational rehabilitative decisions (VRD) after medical Check-ups, stratified by adequacy of communication to the employer

VRD	Communicated to employer	All individuals	SWS-Orig and SWS-SO^#^	SWS-SO 1–3
		*n* (%)	*n* (%)	*n* (%)
**Adequate**				
No rehab needed	Not needed	287 (43)	249 (44)	0 (0)
Rehabilitation accepted	Actively accepted	40 (6)	39 (7)	39 (12)
Employee no longer exposed	Not needed for the individual	6 (1)	5 (1)	5 (2)
Alternative follow-up	Communicated	38 (6)	30 (5)	30 (9)
Already known HAVS	Known by employer	6 (1)	5 (1)	5 (2)
**Semi-adequate**				
Medical investigation commenced	Not communicated	75 (11)	56 (10)	56 (17)
Shorter period until next MCV	Communicated	48 (7)	44 (8)	44 (14)
**Inadequate due to patient**				
Rehabilitation declined	Actively declined	31 (5)	28 (5)	28 (9)
**Inadequate due to physician**				
Individual recommendations only	Not communicated	70 (11)	63 (11)	63 (20)
No action despite findings	Not communicated	58 (9)	53 (9)	53 (16)
Total		660 (100)	572 (100)	323 (100)

^#^ Data on SWS-grading by both occupational physician (SWS-Orig) and semi-objectively (SWS-SO)

Among the 388 individuals without semi-objective findings (SWS-SO 0–1), 382 (98% of the SWS-SO 0–1) were also graded as SWS-Orig 0–1, hence correctly graded (Table 6). Among the 162 individuals with SWS-SO 1, an Inadequate VRD was taken for 68 individuals (42%).

Among the 184 with semi-objective findings (SWS-SO 2–3), 108 individuals (59% of the SWS-SO 2–3) were graded as SWS-Orig 0–1 (i.e. underestimated), and Inadequate VRDs were taken for 54%. The Inadequate VRDs were thus 4.5 times higher amongst the individuals when the SWS-gradings were underestimated by the occupational physicians than when correctly graded. In 80% of the 159 inadequate VRDs the decision to refrain from communication could not be traced to the injured worker.

**Table 6:** Accordance between SWS^**#**^ original (SWS-Orig) and semi-objectively (SWS-SO) grading stratified by vocational rehabilitative decision adequacy

	SWS-SO 0–1 (*n* = 388)			SWS-SO 2–3 (*n* = 184)	
Vocational Rehabilitative Decision	SWS-Orig 0–1 (*n* = 382) n (% of total)	SWS-Orig 2–3 (*n* = 6) n (% of total)		SWS-Orig 0–1 (*n* = 108) n (% of total)	SWS-Orig 2–3 (*n* = 76) n (% of total)
Adequate	274 (48)	4 (< 1)		10 (2)	40 (7)
Semi-adequate	41 (7)	1 (< 1)		39 (7)	19 (3)
Inadequate	67 (12)	1 (< 1)		59 (10)	17 (3)

^#^Stockholm Workshop Scale Sensorineural [15]

In a sub-analysis of the VRDs on the 184 individuals who were graded with SWS-SO 2–3 (Table 7). The number of individuals that actively declined a VRD in semi-objective findings increased from 7 to 16 when we reclassified from SWS-Orig to SWS-SO, whereas the increase went from 10 to 60 individuals, i.e. a sixfold increase, when the physician failed to communicate (*Recommendations only* and *No action despite findings*). There was a statistically significant association between physicians’ grading of severe cases (SWS 2–3) and the three-tier inadequacy status of the VRD (*p* < 0.0001) and between the association when the employee and the physician refrained from communication (*p* < 0.0001).

**Table 7:** Accordance between occupational physician’s and semi-objective SWS^**#**^-grading stratified by adequacy of vocational rehabilitative decisions

Vocational Rehabilitative Decision	SWS underestimated *n* (%)	SWS correct *n* (%)	SWS S-O 2–3 *n* (%)
**Adequate**			
No rehab needed	0 (0)	0 (0)	0 (0)
Rehabilitation accepted	3 (3)	23 (30)	26 (14)
Employee no longer exposed	0 (0)	3 (4)	3 (2)
Alternative follow-up	6 (6)	11 (14)	17 (9)
Already known HAVS	1 (1)	3 (4)	4 (2)
**Semi-adequate**			
Medical investigation commenced	21 (19)	14 (18)	35 (19)
Shorter period until next MCV	18 (17)	5 (7)	23 (13)
**Inadequate due to patient**			
Rehabilitation declined	9 (8)	7 (9)	16 (9)
**Inadequate due to physician**			
Individual recommendations only	20 (19)	5 (7)	25 (14)
No action despite findings	30 (28)	5 (7)	35 (19)
Total	108 (100)	76 (100)	184 (100)

^#^Stockholm Workshop Scale Sensorineural [15]

## Discussion

In almost half of the individuals that needed vocational rehabilitation, the Vocational Rehabilitative Decisions (VRD) were Inadequate, i.e. not reported back to the employer. Among those with semi-objective findings, the occupational physicians underestimated the SWS grades in 59%, and the number of underestimated cases of SWS was much higher among the individuals with Inadequate VRDs. Of the Inadequate VRDs, 80% were not reported to the employer because of the physician’s choice.

The optimal outcome of an MCV with symptoms, or findings, is to initiate a process to make sure a correct diagnosis of HAVS is established and informing the employer. It is not common for the final diagnosis of HAVS to be established during the MCV, as it demands that other potential causes for the findings be excluded [19]. However, a finding of a SWS 2–3, either sensorineural or vascular, should result in the physician informing the employer that the individual is considered unfit for working with hand-arm vibrations [11, 19]. The employer is then obliged to adapt the work environment to severely, or completely, reduce the individual’s exposure [4, 9–12]. The workers in this study that were incorrectly graded in a lower risk category were thus excluded from protective measures, risking aggravation of an untreatable injury with further exposure to hand-arm vibrations [4, 9–12].

The amount of MCVs where the SWS SN was erroneously underestimated, despite the physicians noting positive findings in the status, is concerning. It is possible that more training is required to improve the physicians’ level of knowledge on HAVS and SWS-grading. We have no reason to suspect that this is the case only for the physicians in this study (i.e., in this company), as similar findings of previous low quality examination or VRDs from other OHSs were found in copies of medical records from other OHSs where the employers had changed OHS.

In addition to the tendency to underestimate the grading of SWS, there were also differences in what VRDs were taken after examination, mainly regarding how (and if) the results were communicated to the employer. Interestingly, it was amongst the two most inadequate VRDs (*No follow-up despite findings* and *Individual recommendations only*), i.e. those where the physicians refrained from communicating with the employer, that their underestimation of SWS SN was the highest (fourfolded or more). This top tier was followed by a medium tier where the number of underestimated cases was higher than the number of those correctly graded, containing the two Semi-inadequate actions: *Shorter period until next MCV* and *Medical investigation commenced*, as well as *Rehabilitation declined*, i.e. when the employee actively declined a rehabilitative meeting. In the lowest tier, the unproblematic actions: *Rehab accepted*,* Already known HAVS*, and *Employee no longer exposed*, had minimal to no underestimation.

Unfortunately, the present study lacks data on why inadequate VRDs were taken by the physicians and why employees refuse to share results with their employer. We strongly recommend that possible reasons for why this happens should be investigated further.

Noteworthily, there are also some VRDs physicians should consider abstaining from in the “Semi-adequate”-category. From the physician’s perspective, a grading of SWS 2–3 with the outcome *Shorter period until next MCV* is a nudge to the employer that the MCV has revealed something that increases the risk for injury. By giving the employer this data in a document, the physician has at least signalled a problem and broken professional secrecy. If such a document is provided to the employer after every MCV, the lack of one or a shorter period implies a potential problem with HAVS and the lack of a document is itself a break of secrecy, voluntary or not. However, if a rehabilitation meeting is initiated instead, far more could be done as the physician may help the employer to reduce exposure at an earlier stage and to other employees at risk. If one employee has been exposed enough to cause HAVS, the other employees’ work environment will also likely need to be addressed as part of the mandatory systematic work environment improvement, one of the main aims of the provision.

Coherently, *Medical investigation commenced* should also be seen as inadequate when striving to minimize the exposure for all individuals at risk. It is overall advisable to strive for inclusion of the employer as early as possible. As mitigating exposure is often expensive, in both time and money, the physician potentially deals with an employer who might be negative towards the idea, and a reminder that a legally mandatory vocational rehabilitation may be needed. If that is not sufficient, the physician should report the employer to the Swedish Work Environmental Agency. In this study, no such outcome was recorded in any medical records, despite being mandatory and highly adequate in several cases. It is, however, the company that is reported and not the individual. Therefore such an action might not be recorded in the individual’s medical record.

Finally, an OHS that detects too many injured workers at the MCV might risk losing the employer as a customer, which may also be an incentive to underestimate the severity of HAVS.

### Strengths and limitations

The whole study comprised of data from medical records in a clinical setting. This gives the data high ecological validity but may lower precision since the data initially were not primarily collected for research.

Our finding that almost one third of the individuals have semi-objective findings is a very high number and might be a result of a selection bias, i.e. the comparatively few individuals that attend an MCV are not a representative sample of the population that should be offered an MCV. However, if so, this does not influence the conclusions.

We graded sensory perception loss as positive with only one examination method having findings above the threshold in Table 1. This is an older [15] and somewhat more liberal criterion for SWS-grading than later proposed by Poole et al. [6]. However, we believe this is justified by the intent of the provision, i.e. to find workers exposed to vibrations with incipient HAVS, not necessarily diagnosed. A worker will then have time to work out a personalized plan aiming to minimize the risk of getting a full blown HAVS.

The semi-objective SWS scale in this study lacks the criterion “numbness and tingling”, which is supposed to arise before any neurological findings as the scale is progressive and symptoms should be present at all stages above SWS 0 [15]. However, that information was not obtainable from the medical records.

We used SWS-Orig to set the grade for SWS-SO when there was no data on results from semi-objective testing. By doing so, we may have missed some workers where the physician erroneously underestimated SWS SN. Thus, the number of workers with inadequate VRDs might have been even larger.

We decided to focus on SWS Sensorineural and not include the SWS Vascular due to the large amount of missing data and the lack of objective data on finger blanching.

## Conclusions

Occupational physicians did not SWS-grade 1/8 of the patients and underestimated the number of individuals with SWS 2–3 compared with semi-objective findings. The underestimation was largest (sixfold) in the most severe stratum (SWS 3). We found ten different VRDs in our analysis, five where there were adequate communication with the employer (*No rehab needed*, *Rehabilitation accepted*, *Employee no longer exposed*, *Alternative follow-up*, and *Already known HAVS*), two with semi-adequate communication (*Medical investigation commenced* and *Shorter period until next MCV*), and three when the communication with the employer was absent (*Rehabilitation declined*, *Individual recommendations only*, and *No action despite findings*). The occupational physicians made the largest underestimation of SWS for the VRDs where there was inadequate communication with the employer. Lack of communication with the employer excludes affected workers from the employer’s mandatory protective measures and risks aggravation of an untreatable injury, both for the individual and their similarly exposed colleagues. Further studies on why physicians systemically underestimate the grading of SWS and fail to communicate despite findings, as well as why employees refuse to share their results with their employer, are needed.

## Data Availability

The complete and detailed individual data of all subjects cannot be publicly available for ethical and/or legal reasons due to compromising patient privacy based on Swedish law. The National Ethical Committee (https://etikprovningsmyndigheten.se/en/) has imposed these restrictions. Data can be obtained after application and approval of the research project by the National Ethical Committee and by the data safety committee of Lund University, Sweden.

## Abbreviations

HAVS: Hand-Arm Vibration Syndrome
MCV: Medical Check-ups in work-life for Vibrations
OHS: Occupational Healthcare Service
SWS SN: Stockholm Workshop Scale SensoriNeural
SWS-Orig: Sensorineural SWS grading, original (by the occupational physician)
SWS-SO: Sensorineural SWS grading, semi-objective (as in Table 1)
SWS: Stockholm Workshop Scale
VRD: Vocational Rehabilitative Decisions
